# 尼古丁及尼古丁乙酰胆碱受体在肺癌发生发展过程中的作用

**DOI:** 10.3779/j.issn.1009-3419.2011.09.10

**Published:** 2011-09-20

**Authors:** 志浩 吴

**Affiliations:** 300052 天津，天津医科大学总医院，天津市肺癌研究所，天津市肺癌转移与肿瘤微环境重点实验室 Tianjin Key Laboratory of Lung Cancer Metastasis and Tumor Microenvironment, Tianjin Lung Cancer Institute, Tianjin Medical University General Hospital, Tianjin 300052, China

肺癌是目前世界上最常见的癌症之一，约占所有癌症病例的15%，而吸烟是目前公认的引起肺癌的主要危险因素之一，在发展中国家，约80%的肺癌病例与吸烟有关^[1]^。肺癌的预后总体上仍然不是很乐观，男性的5年生存率为6%-14%，女性为7%-18%^[2]^。在香烟烟雾约4, 000多种化学物质中，已有60多种被确定为致癌物，它们大多通过与DNA相互作用，导致DNA加合物的形成，从而引起细胞中遗传物质或基因的改变。尼古丁是烟草的主要成分之一，其作用主要是导致吸烟者的成瘾性。

尼古丁对中枢神经系统的作用主要是通过尼古丁乙酰胆碱受体（nicotinic acetylcholine receptors, nAChR）实现的。除了中枢神经系统的神经元，全身许多细胞如淋巴细胞、巨噬细胞、树突细胞、脂肪细胞、角质细胞、内皮细胞及肠道和肺的上皮细胞均表达nAChR。尼古丁及其受体nAChR在肺癌的发生发展中具有重要作用，包括调控细胞增殖、细胞凋亡、新生血管的形成及肿瘤的侵袭等^[3]^。因此，本文将对尼古丁在肺癌及其演进过程中所扮演的角色作一综述。

## 1 尼古丁及其受体nAChR

尼古丁的多种功能特性是通过其与各种类型nAChR的相互作用而形成的。nAChR是由各种不同亚单位组成的同源及异源性五聚体构成（α1-10, β1-4）的^[4, 5]^。尼古丁是一种非选择性nAChR激动剂，nAChR五聚体中的β2或β4亚单位与其对尼古丁的亲和力高低有关。尼古丁可激活多种nAChR亚型，作用于交感和副交感神经系统，引起一系列反应。nAChR的上调或下调可对心血管系统、呼吸系统、内分泌系统及中枢神经系统产生生理学效应^[6-8]^。其中研究最多的是香烟的成瘾性效应^[9, 10]^。同其它成瘾性药物一样，尼古丁可使大脑中阿肯柏氏核多巴胺水平增高，进而加强药物的利用或消耗^[11]^。

## 2 肺组织中的nAChR

1997年发现正常人支气管上皮细胞（bronchial epithelial cells, BEC）表达α3-、α4-、α5-及α7-四种nAChR亚单位，这4个亚单位形成钙离子调控通道从而调节细胞的粘附和运动^[12]^。之后又发现在BEC中尼古丁的结合位点具有饱和性。RT-PCR、原位杂交等技术证实了BEC中α7-nAChR的存在^[13]^。值得关注的是，West等^[14]^发现起源于大气道的BEC和小气道上皮细胞（small airway epithelial cells, SAEC）的BEC，两者的nAChR类型有略微不同。BEC选择性表达α3-和α5-nAChR亚单位，而SAEC选择性表达α2-和α4-亚单位，两者都表达α7-、α10-、β2-和β4-亚单位。Plummer等^[15]^研究表明在正常肺组织细胞和肺癌细胞（包括鳞癌、类癌、腺癌、大细胞癌和小细胞癌）中都广泛表达α7-nAChR。由此推断，肺上皮细胞可能广泛表达α7-nAChR，这也提示它在肺组织生物学中的重要作用。

## 3 尼古丁及nAChR对正常肺组织的作用

近年来，随着肺组织细胞中尼古丁乙酰胆碱受体nAChR的发现，胆碱类药物在肺癌发展过程中的作用得到了更多的研究^[16, 17]^。吸烟人群中，血浆尼古丁水平的峰值在白天可达到200 nM左右，在夜晚入睡时则可降至5 nM-10 nM，而直接暴露于烟雾的肺气道的尼古丁水平可升至5倍-10倍，而且峰值和最低值更为明显^[19]^。这样高的水平足够激活α4β2-nAChR^[18, 19]^，而且也可能激活α7-nAChR^[20]^。

粘膜下腺体的上皮细胞中大量表达α7-nAChR。吸烟可明显增加nAChR的相对数量，而这一效应可在10 μM尼古丁处理的BEC培养液中被复制^[15]^。Lam等^[21]^最近发现100 nM尼古丁处理BEC细胞72 h后和144 h后，α1-、α5-、α7-nAChR的RNA水平在72 h明显增加，而撤去尼古丁后，又回到基础水平。为了更深入地探讨这些分子机制，有研究^[22]^采用5 μM尼古丁分别处理BEC 4 h、8 h、10 h后，通过微阵列-生物信息学分析尼古丁所诱导的基因表达图谱发现，在所分析的1, 800个基因中，260个基因明显上调，17个基因明显下调。

用尼古丁处理兔肺上皮细胞不同时间发现，细胞内多条通路的活化状况也不同^[23]^。其中，蛋白激酶C（protein kinase C, PKC）和磷脂酰肌醇3-激酶（phosphatidyl-inositide 3 kinase, PI3K）在尼古丁处理后可暂时激活。Ras及其下游效应分子细胞外信号调节激酶1/2（extracellular signal-regulated kinase, ERK1/2）在长时间暴露于尼古丁后也可激活。尼古丁激活Ras是氨甲蝶林（methotrexate, MTX）所介导的G_1_期检测点受到干扰及活性氧族产生增加的原因^[23]^。这些数据表明持续暴露于尼古丁可干扰G_1_期检测点，并且可通过活性氧族的产生引起DNA损伤。尼古丁激活的多条信号通路中研究最成熟最广泛的是PI3K/Akt信号通路。尼古丁可在几分钟内激活Akt，在45 min-50 min达到高峰，且可以维持数小时。吸烟的肺癌患者体内磷酸化Akt的存在提示尼古丁激活Akt不仅局限在原代培养细胞，而且存在于肺癌细胞。而一旦被尼古丁激活，Akt就可使多个调控细胞周期和蛋白翻译的下游分子发生磷酸化，如糖原合成酶-3（glycogen synthase kinase-3, GSK-3）、真核翻译起始因子4E（eukaryotic translation initiation factor, 4EBP-1）及核糖体激酶p70^S6K[14]^。肺神经内分泌细胞（pulmonary neuroendocrine cells, PNEC）是气道上皮细胞中极为特殊的一部分，它们含有并且可以分泌生物胺，尤其是5-羟色胺（5-hydroxytryptamine, 5-HT）和各种多肽^[24, 25]^。通过分析游离的兔气管中5-HT的释放，发现PNEC的分泌活性是通过尼古丁受体介导的^[26]^。并且尼古丁受体诱导的5-HT的释放同样可存在于体外培养的PNEC细胞中^[27]^。Roman等^[28]^研究发现，暴露于尼古丁的小鼠其纤连蛋白mRNA和蛋白的水平均增高。免疫组化的结果也表明尼古丁与肺泡、气道上皮及血管中纤连蛋白蛋白水平的增高有关。以上数据提示尼古丁通过刺激α7-nAChR依赖的信号通路诱导成纤维细胞产生纤连蛋白，从而导致肺细胞基质成分发生改变。尼古丁可能通过此种机制促进气道周围及肺实质的组织重塑，这也可能是烟草会导致肺功能异常的机制之一。

## 4 尼古丁作为致癌物

Hecht等^[29]^假设尼古丁是经2'-羟基进行代谢的，它最终生成酮酸和羟基酸作为尿液新陈代谢的产物。虽然这条通路在哺乳动物中未建立起来，但它仍然具有重要的意义，因为尼古丁的产物，2'-羟基, 4-(甲胺基)-1-(3-吡啶)-1-丁酮(氨基酮)，可转变为烟草-特异性的肺部强致癌物4-(甲基亚硝胺)-1-(3-吡啶)-1-丁酮。将尼古丁与细胞色素P450 2A6共同孵育可产生氨基酮，用氨基酮做媒介，将人肝细胞微粒体与尼古丁共同孵育则可产生酮酸。最近有文献^[30]^对与尼古丁结构相似的致癌物亚硝胺4-(甲基亚硝胺)-1-(3-吡啶)-1-丁酮(nitrosamines 4-(methylnitrosamino)-1-(3-pyridyl)-1-butanone, NNK)和N'-亚硝基去甲基烟碱（N'-nitrosonornicotine, NNN）的作用机制做了阐明。NNK对α7-nAChR具有高度亲和性，用NNK或尼古丁处理小细胞肺癌（small cell lung cancer, SCLC）或PNEC，可使α7-nAChR的表达增加，引起钙离子内流，激活PKC、Raf-1、ERK1/2和c-myc，导致细胞增殖。NNK或NNN处理支气管肺泡细胞BEP2D后，细胞增殖潜能增强，并且可被α-BTX阻断，同时，增殖细胞核抗原（proliferating cell nuclear antigen, PCNA）和凋亡抑制蛋白*Bcl-2*基因表达成倍增加。NNK激活nAChR可导致转录因子GATA-3、核因子κB（nuclear factor-κB, NF-κB）及信号转导和转录激活因子-1（signal transducer and activator of transcription, STAT-1）的激活，而NNN主要激活GATA-3和STAT-1。这些数据均表明NNK和NNN的功能是通过nAChR实现的，并且nAChR拮抗剂有可能成为肿瘤化学预防的药物^[31]^。

## 5 尼古丁及其受体nAChR在肺癌中的作用

肺癌从组织学上主要分为：类癌、SCLC和非小细胞肺癌（non-small cell lung cancer, NSCLC）。NSCLC又分为：腺癌（adenocarcinoma, AD）、鳞癌（squamous cell carcinoma, SQ）及大细胞癌。腺癌是男女性共发的最常见的组织学类型，但与男性相比，女性中腺癌更为常见，而鳞癌则较少。香烟烟雾与上述几种组织亚型均有关联，但是吸烟者最容易发展为鳞癌。

SCLC和NSCLC细胞系均存在α7-nAChR^[32]^。研究^[21]^表明，非吸烟肺癌患者体内α6-和β3-nAChR的表达水平较吸烟患者高。最近，全基因组相关性研究（genome-wide association studies, GWAS）表明人类染色体15q24-25与肺癌的高风险具有显著关系^[33]^。通过单核苷酸多态性（single nucleotide polymorphisms, SNPs）分析发现，染色体15q24-25区域包含有编码α3-、α5-及β4-nAChR的基因簇CHRNA5、CHRNA3及CHRNB5^[33, 34]^，并且，α5-α3-nAChR与吸烟相关性肺癌具有特异性，而与不吸烟性肺癌及其它吸烟相关性癌症如膀胱癌和肾癌无关。此外，最新的研究^[35]^还表明，除了肺癌，α9-nAChR在与吸烟相关的人乳腺癌中也存在过表达，且尼古丁可通过上调α9- nAChR的表达诱导正常乳腺上皮细胞MCF-10A的转化。

目前发现尼古丁在肺癌细胞（SCLC和NSCLC）中的主要作用为促进肿瘤细胞增殖和抑制药物诱导的细胞凋亡。A549细胞同其它肺腺癌细胞一样，存在一个有活性的胆碱能系统，包括Ach、胆碱乙酰转移酶（choline acetyltransferase, ChAT）、积聚有胆碱的膜囊泡、乙酰胆碱酯酶（acetylcholinesterase, AChE）及nAChR^[36]^。将A549细胞接种到免疫缺陷小鼠NOD/SCID，发现尼古丁可明显促进肿瘤的生长，而尾静脉注射一定量的尼古丁（0.6 mg/kg， > 15 d），则可降低细胞凋亡（TUNEL分析）、增加血管的数量（CD31阳性细胞百分比）及增加Ki67的表达。Ki67是肿瘤细胞增殖的标记物，可反映肿瘤内磷酸化AKT的水平^[37]^。以上这些数据均表明尼古丁在肿瘤进展中具有一定的作用。尼古丁在肿瘤发展中的作用不仅与新生血管数量的增加有关，而且可能参与细胞死亡和增殖的调控。

### 5.1 细胞增殖方面

有研究^[38]^表明，在仓鼠中单独的尼古丁可能并不会导致肺癌的发生，而尼古丁伴有缺氧则可以诱发肺癌，从而提示长期的nAChR的活化伴有缺氧损伤可引起肿瘤的发生。在NSCLC中，尼古丁可以靶向表皮生长因子（epidermal growth factor, EGF）的释放，导致EGF结合到其受体EGFR，并激活Ras-Raf-ERK信号通路，从而导致细胞增殖^[39, 40]^。Ras-Raf-ERK信号通路可被α7-nAChR拮抗剂α-银环蛇毒素（α-bungarotoxin, α-BTX）和α-环磷酰胺（α-cobratoxin, α-CTX）所阻断^[41]^。此外，β-arrestin信号通路也可参与尼古丁诱导的细胞增殖^[42]^。β-arrestin结合到α7-nAChR可激活Src和Raf通路，Raf又结合到Rb，Rb的失活最终导致细胞进入S期。这一通路可被α-BTx及另外一种α7-nAChR拮抗剂MLA（methyllycaconitine）阻断。另外，尼古丁还可激活PI3K及哺乳动物雷帕霉素靶蛋白（manmal target of rapamycin, mTOR），导致细胞增殖^[43]^。

### 5.2 细胞凋亡方面

尼古丁通过激活PI3K/Akt直接使Bax磷酸化从而使其凋亡的功能失活^[44, 45]^。尼古丁还可引起Akt下游底物如GSK-3、p70^S6K^、4EBP-1的磷酸化参与细胞凋亡的抑制^[14]^。Akt的激活还可使凋亡抑制蛋白Survivin和XIAP水平上调，从而在尼古丁抗凋亡活性中发挥关键作用^[46]^。尼古丁通过上调NF-κB发挥其抗凋亡的作用^[47]^。

### 5.3 新生血管形成方面

Heeschen等^[48]^从解剖结构及功能上证实了尼古丁可以诱导血管形成。同时，他们也证明了尼古丁可加速肿瘤和动脉粥样斑的生长，并伴有新生血管形成的增加。在老鼠后肢缺血模型中，尼古丁可促进毛细血管及其附属物的生长，促进组织的灌注。尼古丁的以上这些效应都是通过nAChR介导的，内皮细胞产生的氮氧化物、前列腺素及血管内皮生长因子（vascular endothelial growth factor, VEGF）都有可能在这些效应中发挥作用。有研究^[49]^表明，尼古丁作为二手烟（second-hand smoke, SHS）的主要成分之一，与血浆中一些血管源性细胞因子如VEGF的增加有关，而SHS的血管效应可被内皮细胞的nAChR抑制剂所阻断。此外，有研究^[50]^表明，尼古丁还可通过刺激缺氧诱导因子（hypoxia inducible factor, HIF-1α）的积聚而调控新生血管的形成。

### 5.4 肿瘤侵袭和转移方面

上皮细胞间质化（epithelial-mesenchymal transition, EMT）与细胞运动能力及侵袭能力的增加有密切关系，也是目前肿瘤转移研究的热点之一。尼古丁可引起上皮细胞的标记物E-钙粘蛋白（E-cadherin）、β-连环蛋白（β-catenin）及紧密结合蛋白ZO-1的表达下降，同时间质细胞标记物纤连蛋白和波形蛋白（vimentin）的表达增加^[51, 52]^。此外，尼古丁可通过激活与人肺癌细胞迁移和侵袭有关的PKC诱导μ-和m-钙激活酶（calpains）的磷酸化，从而激活细胞运动的信号^[53]^。在体内，PKC可直接磷酸化μ-和m-钙激活酶，PKC的过表达可导致μ-和m-钙激活酶磷酸化的增加。尼古丁还可诱导c-Src的活化，它是PKC的上游激酶。用α-BTX处理细胞，可阻断尼古丁诱导的钙激活酶的磷酸化并抑制钙激活酶的活性，抑制创伤修复、细胞迁移和侵袭，这表明尼古丁诱导钙激活酶的磷酸化在一定程度上是通过α7-nAChR实现的。通过RNA干扰技术靶向沉默PKC的表达可抑制尼古丁诱导的钙激活酶的磷酸化及其活性，抑制细胞的迁移和侵袭，表明PKC是尼古丁介导的细胞运动的必要组成部分。此外，尼古丁还可诱导μ-和m-钙激活酶从肺癌细胞中分泌到培养基中，这有可能导致细胞外基质（extracellular matrix, ECM）中底物的裂解。以上这些发现揭示了PKC通过钙激活酶的激活，在人肺癌细胞的迁移和侵袭中所扮演的新角色。

## 6 结语

尽管没有证据表明尼古丁本身可以诱导肺癌，但是众多研究证实尼古丁可以促进体内癌细胞的生长，促进内皮细胞的增殖，其中，尼古丁乙酰胆碱受体nAChR起着重要的介导作用，尼古丁通过激活不同的nAChR亚型发挥其在肺癌中的重要作用。通过抑制nAChR的蛋白水平可明显消除尼古丁及NNK在肿瘤模型中诱导细胞增殖的作用。这些发现将有助于我们更好地研究和理解尼古丁及nAChR在肺癌发生发展中的作用。目前，已有一些nAChR特异性的激动剂及拮抗剂用于多种疾病治疗的临床试验（癌症除外），因此，特异性靶向肿瘤中过表达的nAChR亚型的拮抗剂可能会为今后肿瘤的治疗提供一种新的途径^[54]^。
